# Hot-Water Injections *Vice* Nutrient Enemata

**Published:** 1904-06-11

**Authors:** 


### HOT-WATER INJECTIONS *VICE* NUTRIENT ENEMATA.

DR. W. PASTEUR[1] points out the value of rectal injections of water at the body temperature as a substitute for the ordinary nutrient enemata in cases of gastric ulcer. That by the latter method patients can be kept alive and in comparative comfort for several weeks is admitted. Comfort and relief from pain are prompt, but nutrient enemata also produce certain unfortunate consequences. One of these is thirst. To permit ice or small quantities of water by the mouth is to lose some measure of the physiological rest prescribed for the stomach. Another consequence of the use of nutrient enemata, at least when they contain beef-tea, is the development of an unpleasant taste of beef-tea in the mouth and of nausea. Enemata containing 10 oz. of plain water instead of the smaller nutrient enemata generally employed avoid all the above symptoms. Further, the patients are much more comfortable than under the latter *régime*, and their recovery is quite as rapid. As a rule, it is found that small quantities of peptonised milk can be given by the mouth in the course of a week, but in several instances nothing has been given for 10 to 14 days without other than good results.

[1] Lancet, May 21, 1904.

